# mRNA Profile in Milk Extracellular Vesicles from Bovine Leukemia Virus-Infected Cattle

**DOI:** 10.3390/v12060669

**Published:** 2020-06-20

**Authors:** Hinata Ishikawa, Md. Matiur Rahman, Marika Yamauchi, Shigeo Takashima, Yoshiko Wakihara, Yuji O. Kamatari, Kaori Shimizu, Ayaka Okada, Yasuo Inoshima

**Affiliations:** 1 Laboratory of Food and Environmental Hygiene, Cooperative Department of Veterinary Medicine, Gifu University, 1-1 Yanagido, Gifu 501-1193, Japan; s8025001@edu.gifu-u.ac.jp (H.I.); matiur.vetmed@gmail.com (M.M.R.); r8023025@edu.gifu-u.ac.jp (M.Y.); skaori@gifu-u.ac.jp (K.S.); okadaa@gifu-u.ac.jp (A.O.); 2 The United Graduate School of Veterinary Sciences, Gifu University, 1-1 Yanagido, Gifu 501-1193, Japan; 3 Department of Medicine, Faculty of Veterinary, Animal and Biomedical Sciences, Sylhet Agricultural University, Sylhet 3100, Bangladesh; 4 Division of Genomics Research, Life Science Research Center, Gifu University, 1-1 Yanagido, Gifu 501-1193, Japan; staka@gifu-u.ac.jp (S.T.); wakihara@gifu-u.ac.jp (Y.W.); 5 Division of Instrumental Analysis, Life Science Research Center, Gifu University, 1-1 Yanagido, Gifu 501-1193, Japan; kamatari@gifu-u.ac.jp; 6 Education and Research Center for Food Animal Health, Gifu University (GeFAH), 1-1 Yanagido, Gifu 501-1193, Japan; 7 Joint Graduate School of Veterinary Sciences, Gifu University, 1-1 Yanagido, Gifu 501-1193, Japan

**Keywords:** bovine leukemia virus, mRNAs, milk extracellular vesicles, profile

## Abstract

Milk extracellular vesicles (EVs) form an excellent source of mRNAs, microRNAs (miRNAs), proteins, and lipids that represent the physiological and pathological status of the host. Recent studies have reported milk EVs as novel biomarkers for many infectious diseases in both humans and animals. For example, miRNAs in milk EVs from cattle were used for early detection of bacterial infection in the mammary gland. Based on these findings, we hypothesized that mRNAs in milk EVs are suitable for gaining a better understanding of the pathogenesis of bovine leukemia virus (BLV) infection and prognosis of the clinical stage in cattle. For that purpose, milk EVs were isolated from BLV-infected and uninfected cattle, and mRNAs were investigated using microarray analysis. Gene ontology (GO) and Kyoto Encyclopedia of Genes and Genomes (KEGG) pathway analyses were performed mainly focusing on the differentially expressed genes (DEGs) in milk EVs from BLV-infected cattle. GO and KEGG analyses suggested the DEGs in milk EVs from BLV-infected cattle had involved in diverse molecular functions, biological processes, and distinct disease-related pathways. The present study suggested that BLV infection causes profound effects on host cellular activity, changing the mRNA expression profile in milk EVs obtained from BLV-infected cattle. Overall, our results suggested that the mRNA profile in milk EVs to be a key factor for monitoring the clinical stage of BLV infection. This is the first report of mRNA profiling of milk EVs obtained from BLV-infected cattle.

## 1. Introduction

Extracellular vesicles (EVs) are small membranous particles and are extracellularly secreted from a wide variety of mammalian cells [1]. EVs are present in all biological fluids, including plasma, malignant effusions, urine, saliva, breast milk, and bronchoalveolar lavage fluid [2]. There is still some variability in the different classes of EVs. However, there is a common perception about the classes of EVs that included exosomes, ectosomes, or shedding microvesicles, apoptotic bodies, and other EVs subsets which were differentiated according to their size, biogenesis, and releasing pathway [3]. Among EVs, exosomes, the size ranges from 30 to 150 nanometer in diameter are of particular interest [1]. According to minimal information for studies of extracellular vesicles guidelines 2018 were urged to consider the use of an assigning concept as EVs [4]. EVs contain microRNAs (miRNAs), mRNAs, lipids, and cellular proteins that play vital roles in intercellular communication [5]. For example, EVs from cancer cells are known to contribute to the horizontal propagation of oncogenes and to tumor microenvironment, thus acting as possible prognostic biomarkers of cancers [6]. A recent study had revealed a number of mRNAs from healthy porcine milk EVs to be closely related to metabolic, degradation, and signaling pathways [7]. Another study had reported mRNAs in milk EVs of cattle origin to perform important physiological and immunological functions [8]. Sun et al. [9] had reported miRNAs such as bta-miR-142-5p and bta-miR-223 in milk EVs from cattle to act as potential biomarkers for early detection of bacterial infection in the mammary gland. Based on these reports, we predicted mRNAs in milk EVs from bovine leukemia virus (BLV)-infected cattle to possibly be useful for the detection of clinical status and pathological conditions. 

BLV is a member of the genus Deltaretrovirus in the family Retroviridae, and also a causative agent of enzootic bovine leukosis (EBL), which is characterized by B-cell lymphosarcoma [10]. EBL is currently one of the most commonly reported neoplastic disease in cattle, being detected worldwide and causing serious economic loss all over. In many cases of BLV infection, the infected cattle remain as life-long virus carriers without manifesting clinical signs [11]. BLV is mainly transmitted horizontally by direct exposure to biological fluids contaminated with BLV-infected lymphocytes, such as during inappropriate reuse of injection needles and gloves for rectal examination, and also from bites of hematophagous insects [12,13,14]. There is no vaccine or treatment for EBL yet, and precursory clinical signs of EBL are still unknown [15]. In Europe, few countries such as Finland, England, and Denmark have successfully eradicated EBL by nationwide detection test and slaughter of BLV-infected cattle [16]. In Japan, however, slaughter of all BLV-infected cattle is impossible, since a national serological study had revealed the presence of antibodies against BLV in 40.9% and 28.7% of dairy and beef cattle, respectively [15]. Additionally, the number of cattle notified as having EBL has increased gradually in Japan (https://www.maff.go.jp/j/syouan/douei/kansi_densen/kansi_densen.html). After being diagnosed with EBL, the cattle and relevant products are not legally eligible for human consumption, in Japan, owing to the policy that mandates meat and milk products be supplied only from healthy cattle.

Thus, the objective of our present study was to identify the mRNA profile in milk EVs derived from BLV-infected cattle. This study provided robust information regarding the mRNA profile that could serve as a useful benchmarking resource for the development of biomarkers in subsequent investigations on BLV infection in cattle. This is the first comprehensive research focused on mRNA profile in milk EVs from BLV-infected cattle.

## 2. Materials and Methods

### 2.1. Hematology

Blood samples of 10 milliliters (ml) of each of the 16 Holstein cows were collected from two different farms and directly allocated to vacuum blood collection tubes with or without an anti-coagulant (VP-AS076K, VP-NA050K, and VP-H070K, Terumo, Tokyo, Japan). Total white blood cells (WBCs) and lymphocyte counts were measured by an automatic cell counter Celltac α (Nihon Kohden, Tokyo, Japan). The increased lymphocyte count was checked based on the European Community’s leukosis key [17]. After WBCs and lymphocyte counts, 1.3 mL of each of the anticoagulated blood samples were centrifuged at 2500× *g* for 15 min at 25 °C for plasma separation by a centrifuge, MAX-307 (Tomy Seiko, Tokyo, Japan). Plasma samples were collected from the top portion of the tube and used for lactate dehydrogenase (LDH) isozymes measurement later. DNA was extracted from 300 microliter (µL) of the bottom layered with buffy coat. 

#### 2.1.1. Detection of Serum Antibodies against BLV

Serum was separated from blood by centrifugation at 3000× *g* for 15 min at 25 °C by using a centrifuge, MAX-307. Levels of anti-BLV antibodies in serum were measured using an anti-BLV antibody enzyme-linked immunosorbent assay (ELISA) kit (JNC, Tokyo, Japan) according to the manufacturer’s instructions. 

#### 2.1.2. Detection of BLV Provirus

WBC was isolated by hemolysis of red blood cell with 0.83% ammonium chloride followed by washing twice with phosphate buffer saline (PBS). Total DNA was extracted from WBCs by using QIAamp DNA Mini Kit (51304, Qiagen, Hilden, Germany) according to the manufacturer’s instructions. After measurement of DNA concentration of WBC DNA by a spectrophotometer Nano Drop Lite (Thermo Fisher Scientific, Waltham, MA, USA). Primers to amplify the envelop or pX region of BLV were used for nested polymerase chain reaction (PCR) according to the protocol of Fechner et al. [18] and Murakami et al. [19]. PCR was carried out in a total reaction volume of 20 µL containing 0.5 U of polymerase from Go Taq Hot Start Green Master Mix (M5122, Promega, Madison, WI, USA) or Sapphire Amp Fast PCR Master Mix (RR350A, Takara Bio, Kusatsu, Japan), 0.5 µM of forward and reverse primers, and 1 µL of extracted WBC DNA (100 to 400 ng). Thermal cycling condition was as follows: 95 °C for 2 min, followed by 35 cycles of 94 °C for 45 s, 62 °C for 30 s, 72 °C for 30 s, and finally 72 °C for 4 min.

#### 2.1.3. Measurement of BLV Proviral Load 

BLV-infected cattle with high proviral load (HPL) in blood were selected for this study. It was reported that BLV-infected cattle with HPL in blood were considered as cattle at high risk to be BLV spreaders and might be one of the factors of disease progression [20]. BLV proviral load was measured by using 100 ng of WBC DNA by real-time PCR (qRT-PCR). The amplification was carried out in a reaction mixture containing 12.5 µL of 2× CycleavePCR Reaction Mix (CY510, Takara Bio), 5 µL of probe/primer/positive control for BLV (CY415, Takara Bio), 5 µL of a template DNA sample, and PCR grade water to increase the volume up to 25 µL. For the proviral quantification, BLV tax gene was used as a control from the kit (CY415, Takara Bio) and BLV proviral DNA was measured by a Thermal Cycler Dice Real Time System III (TP970, Takara Bio) according to the manufacturer’s instructions. After the measurement, BLV proviral copies of > 5000/100 ng of WBC DNA was considered HPL in BLV-infected cattle (Table 1). Hematology test, detection of serum antibodies against BLV, detection of BLV provirus, and measurement of BLV proviral load were conducted by the Gifu Central Livestock Hygiene Service Center (Gifu, Japan).

#### 2.1.4. Measurement of LDH Isozymes

Previously, LDH isozyme activity in blood had been reported to reflect disease progression of EBL; especially, increased LDH 2 and 3 percentages were established as a key parameter for the diagnosis of lymphosarcoma [21]. For this reason, the current study focused on LDH isozyme activity in cattle blood. LDH isozymes were measured by a Hydrasys 2 Scan (Sebia, Paris, France) using HYDRAGEL 7 ISO-LDH (Sebia), conducted by a clinical laboratory testing company, Fujifilm Vet Systems (Tokyo, Japan). More than 40% of LDH 2 + 3 was considered as a high LDH (HLDH) count in BLV-infected cattle, whereas less than 25% of LDH 2 + 3 was considered as a low LDH (LLDH) count in uninfected healthy cattle (Table 1). 

### 2.2. Classification of Milk Samples

For comparison of the mRNA profile in milk EVs, we categorized the cattle into two experimental groups, Experiment 1 and Experiment 2. BLV-infected cattle with HPL and uninfected cattle were grouped into Experiment 1. On the other hand, BLV-infected cattle with HPL + HLDH and uninfected cattle with LLDH were grouped into Experiment 2. The clinical status of all cattle used in this study is listed in Table 1. Raw milk samples were collected from these two groups independently. After collection, milk was stored at 4 °C or −80 °C until use. 

#### 2.2.1. EV Isolation

For removing milk fat globules (MFGs), somatic cells, and cell debris, raw milk samples were centrifuged at 2000× *g* for 20 min at 4 °C in an A508-C rotor (Kubota, Tokyo, Japan) using model 7000 centrifuge (Kubota), as described previously with slight modifications [22,23]. Defatted milk was pre-warmed at 37 °C for 10 min, acetic acid (AA) was mixed with the milk [milk/AA = 100 (volume)], and the resulting milk was stirred for 5 min at room temperature, followed by centrifugation at 5000× *g* at 25 °C for 20 min in an R14A rotor (Hitachi Koki, Tokyo, Japan) using Himac CR20GII centrifuge (Hitachi Koki). Casein was pelleted and supernatant (whey) was filtered sequentially through 1.0, 0.45, and 0.2-μm filters (GA-100, C045A047A, and C020A047A, Advantec, Tokyo, Japan). EVs were isolated from 50 mL of whey using two successive ultracentrifugation steps (UC): at 100,000× *g* for 1 h at 4 °C in a P42A angle rotor (Hitachi Koki), and at 100,000× *g* for 1 h at 4 °C in a P42ST swing rotor (Hitachi Koki) using Himac CP60E ultracentrifuge (Hitachi Koki). After the first UC, the supernatant was discarded and pellet was suspended in 8 mL of phosphate buffered saline to transfer it to 13PET tube (Hitachi Koki). The second UC was performed next, and supernatant was discarded. Pelleted EVs were stored at −80 °C for further use. Isolation of EVs was confirmed by detecting EV-surface-marker proteins CD9, CD63, CD81, and MFG-E8 by western blot analysis, as described previously [23].

#### 2.2.2. Transmission Electron Microscopy (TEM) Analysis of Milk EVs

Morphological examination of isolated milk EVs from BLV-infected and uninfected cattle were carried out by TEM as described previously [23] with slight modifications. EVs pellet solution was 10 times diluted with distilled water and applied into glow-discharged carbon support films on copper grids and stained with 2% uranyl acetate, following examined by an electron microscope, JEM-2100F (JEOL, Tokyo, Japan) at 200 kV. 

#### 2.2.3. RNA Extraction and cDNA Synthesis

RNA was extracted from EVs using a Maxwell RSC simply RNA Tissue Kit (AS1340, Promega). The quality of extracted RNA was determined using an Agilent 2100 Bioanalyzer (Agilent Technologies, Santa Clara, CA, USA). 

#### 2.2.4. Microarray Analysis

Microarray probes were prepared from EV RNA, using Low Input QuickAmp Labeling one-color Kit (5190-2305, Agilent Technologies), and were hybridized with Bovine Gene Expression Microarray v2.0, 4x44K (G2519-F-23647, Agilent Technologies). The bovine Microarray v2.0, 4x44K contained 43,668 probes. Hybridized microarray slides were scanned and fluorescence intensities measured using an ArrayScan (Agilent Technologies). The obtained data were analyzed with GeneSpring GX software (Agilent Technologies). The data normalization was performed by 75 percentile shift. The probes ranked in lower than 20 percentiles or with the flag “compromised” in all samples were filtered out. The probes showing the cut off value (CV) of more than 50% in each condition were filtered out. The probes with significant expression changed between the conditions were detected by the moderated *t*-test [24] with Benjamini–Hochberg multiple testing correction [25] and visualized as volcano plots and heat-maps. Corrected *p*-value cut off (i.e., false discovery rate, FDR) of 0.05 was applied.

#### 2.2.5. Gene Ontology (GO) and Kyoto Encyclopedia of Genes and Genomes (KEGG) Pathway Analyses 

GO analysis was conducted for deeper insight into the differentially expressed genes (DEGs) from both Experiments 1 and 2, using online versions of DAVID v6.8 (http://www.david.abcc.ncifcrf.gov/) and PANTHER (http://www.pantherdb.org/) software. KEGG pathway analysis was performed to explore the significant pathways of targeted DEGs; *p*-value was calculated by two-sided hypergeometric test and Benjamini–Hochberg adjustment. A GO term or KEGG pathway with false discovery rate (FDR) <0.05 was considered statistically significant. The enriched GO terms and pathways of DEGs were tabulated by enrichment score [−log_10_ (*p* value)]. 

### 2.3. Ethical Approval

All procedures of blood and milk samples collection in this study were approved by the Gifu University Animal Care and Use Committee (approval number 17046, approved on 4 September, 2017).

## 3. Results

### 3.1. BLV Infection and Clinical Status

BLV infection, hematology, and serum chemistry of cattle were examined and recorded in Table 1. Table 1 indicated the results of the clinical parameters of the animals using in this study. In both experiments 1 and 2, BLV provirus or BLV antibody was checked either by nested PCR or ELISA for the confirmation of presence of BLV infection in cattle. In Experiment 1, four BLV-infected cattle had high copy number of provirus >5000/100 ng of WBC DNA indicating the HPL cattle group. In Experiment 2, another four BLV-infected cattle had also a high copy number of provirus >5000/100 ng of WBC DNA along with LDH 2 + 3 percentage >40% indicating the HPL cattle with HLDH count in BLV-infected cattle. Whereas the LDH 2 + 3 <25% was considered an LLDH count in healthy uninfected cattle. Moreover, in both Experiments 1 and 2, the WBC and lymphocyte counts were very high indicating either ‘Suspect’ or ‘Lymphocytic’ according to the EC key parameter. 

### 3.2. TEM Analysis of Milk EVs

From the TEM analysis, the morphological features of milk EVs from BLV-infected and uninfected cattle were showed a similar spherical bilayer shape (Figure S1). 

### 3.3. Microarray Analysis of Experiments 1 and 2

**Figure 1:**
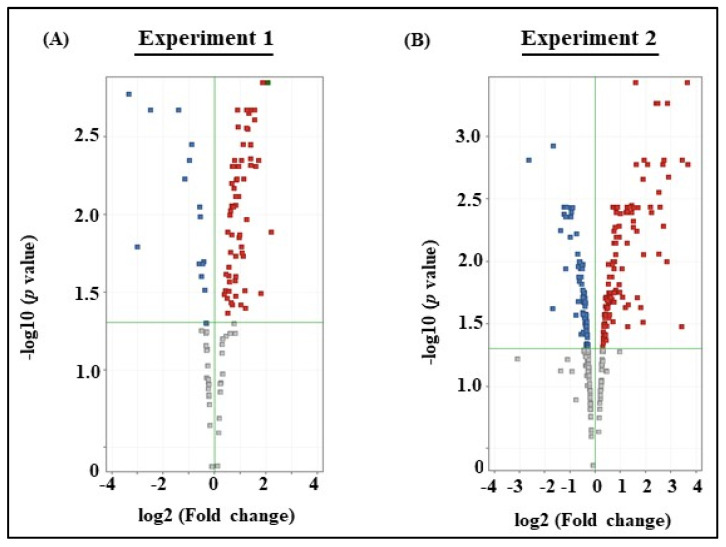
Volcano plot illustrated differentially expressed genes (DEGs) in milk extracellular vesicles (EVs) from BLV-infected cattle with high proviral load (HPL) and uninfected cattle in Experiment 1 (**A**) and BLV-infected cattle with HPL+ high lactate dehydrogenase 2 + 3 (HLDH) and uninfected cattle with low LDH (LLDH) in Experiment 2 (**B**). The up-regulated genes were indicated in red and down-regulated genes were in blue. The fold change (in log_2_ scale) of genes abundance was plotted on the x-axis, with statistical significance expressed as -log_10_ [false discovery rate (FDR)] on the y-axis. Genes not classified as DEGs were plotted in gray.

**Figure 2:**
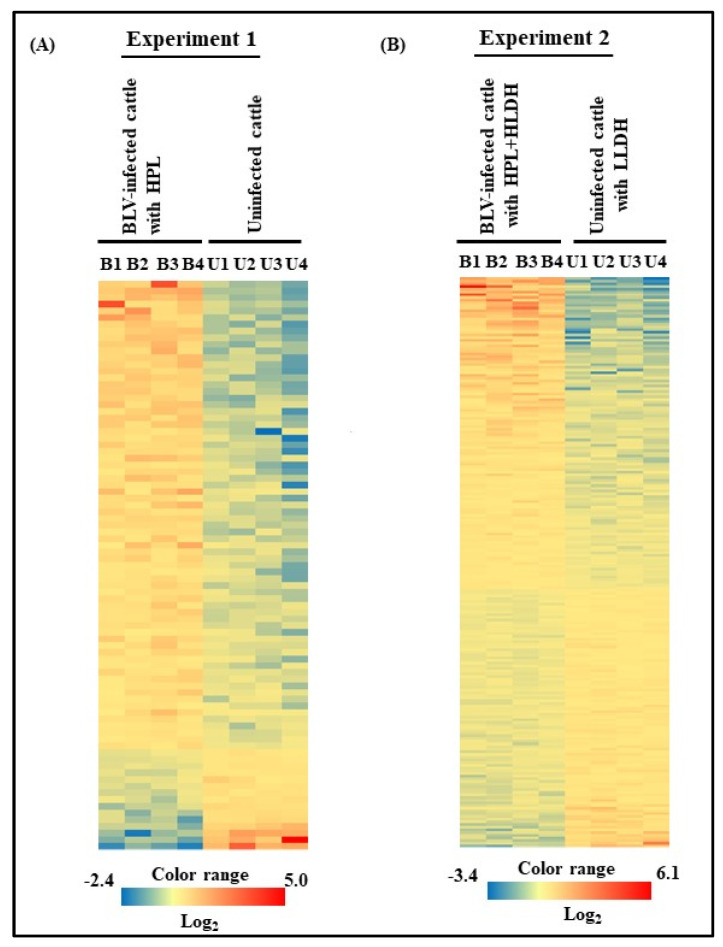
Heat map of DEGs in milk EVs from BLV-infected cattle with HPL and uninfected cattle in Experiment 1 (**A**) and BLV-infected cattle with HPL + HLDH and uninfected cattle with LLDH in Experiment 2 (**B**) were shown. Color-coded scale bar represents the relative content of genes. High level of expression was shown in red and low level was shown in blue.

From Experiment 1, microarray analysis revealed a total of 75 DEGs with statistically significance by *t*-test (*p* < 0.05) in milk EVs from BLV-infected cattle with HPL, as seen from the absolute value of logFc compared to that from uninfected cattle (Table S1). Volcano plot represented the up-regulated and down-regulated genes in milk EVs between BLV-infected cattle and uninfected cattle (Figure 1A). Among them, the top 5 up-regulated and 5 down-regulated genes included laminin gamma 2 (*LAMC2)*, solute carrier family 35 member E4 *(SLC35E4)*, solute carrier family 22 member 17 *(SLC22A17)*, starch binding domain 1 *(STBD1)*, transmembrane protein 255A *(DKK2)*, serpin B9 *(SERPINB9)*, olfactomedin like 2B *(OLFML2B)*, and nucleolar protein 9 *(NOL9)*. Relative content of expression of genes in milk EVs were shown as a heat map (Figure 2A). 

From Experiment 2, microarray analysis revealed a total of 176 DEGs in milk EVs from BLV-infected cattle with HPL + HLDH showed significant difference by *t*-test (*p* < 0.05) as seen by the absolute value of logFc compared to that from uninfected cattle with LLDH (Table S2). Volcano plot represented the significantly up-regulated and down-regulated genes in milk EVs between BLV-infected cattle with HPL + HLDH and uninfected cattle with LLDH (Figure 1B). The top 5 up-regulated and 5 down-regulated genes included myoferlin (*MYOF*), fatty acid elongase 5 (*ELOVL5)*, transmembrane protein 156 (*TMEM156)*, kinesin like protein *(KIF23)*, adenosine monophosphate deaminase 3 (*AMPD3)*, MER proto-oncogene- tyrosine kinase *(MERTK)*, fetuin B *(FETUB)*, heat shock protein family A *(HSPA1B)*, neuronal differentiation 4 *(NEUROD4)*, and uncharacterized gene *(LOC616782)*. Relative content of expression of genes in milk EVs were shown as a heat map (Figure 2B).

### 3.4. GO and KEGG Pathway analyses of Experiments 1 and 2

**Figure 3:**
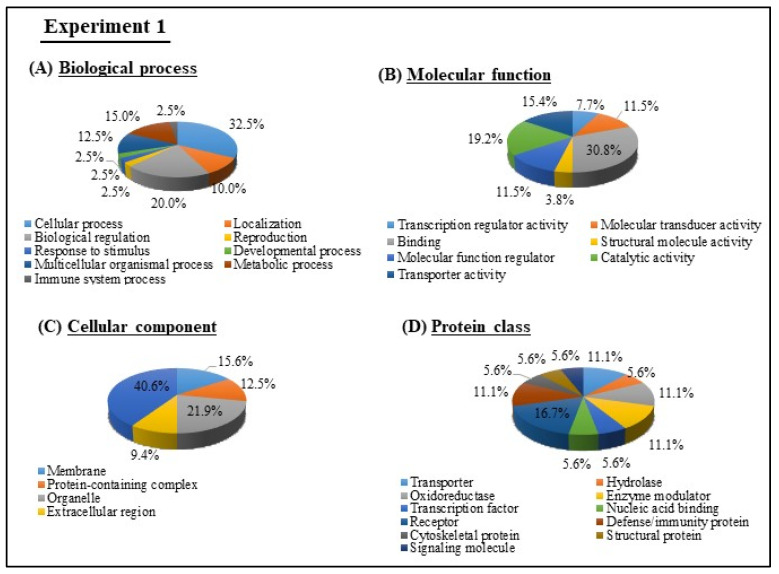
Gene ontology (GO) of DEGs in milk EVs from BLV-infected cattle with HPL in Experiment 1 were analyzed and categorized according to biological processes (**A**), molecular functions (**B**), cellular components (**C**), and protein class (**D**), using PANTHER analysis.

To get functional insights, GO analysis was performed for the 75 DEGs in milk EVs from BLV-infected cattle with HPL from Experiment 1. GO terms of biological processes, molecular functions, cellular components, and protein class of up-regulated and down-regulated genes were determined by PANTHER analysis (Figure 3). The biological processes included mainly cellular process (32.5%), biological regulation (20%), and metabolic process (15%). The most ubiquitous molecular function included protein binding (30.8%) and catalytic activity (19.2%). Cellular components were mostly engaged in cell (40.6%), organelle (21.9%), and membrane (15.6%). The most enriched protein class included receptor (16.7%) and defense/immunity proteins (11.1%). 

**Figure 4:**
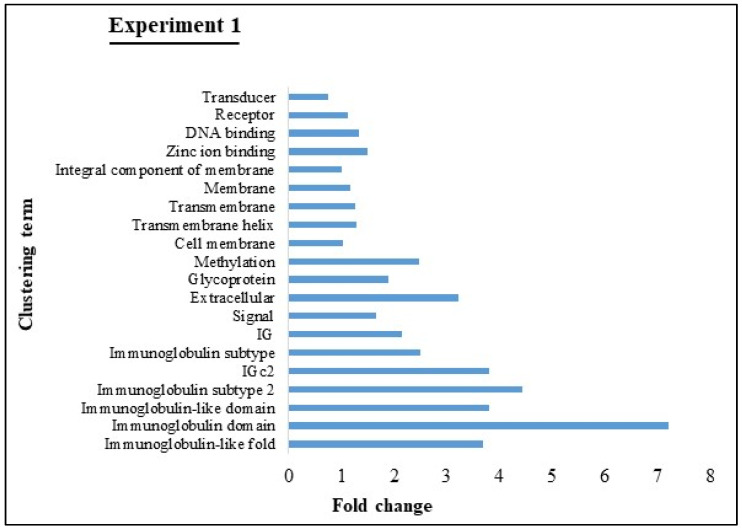
Functional annotation clustering (using DAVID v6.8) of DEGs enriched in milk EVs from BLV-infected cattle with HPL in Experiment 1. (Top 20 with high fold change). X-axis represented fold change and Y-axis represented clustering term.

Functional annotation clustering DEGs in milk EVs from BLV-infected cattle with HPL in Experiment 1 showed notable diversity of biological activities; it identified a total of 6 clusters, such as immunoglobulin domain, immunoglobulin subtype 2, immunoglobulin like fold, immunoglobulin like domain, IGc2, extracellular, and glycoprotein (Figure 4). The KEGG pathway analysis identified 153 distinct pathways of DEGs from Experiment 1 (Table 2). Results showed the top 10 pathways to include cancer pathways, PI3K-Akt signaling pathway, metabolic pathways, MAPK signaling pathway, cell adhesion molecules, Ras signaling pathway, phagosomes, B-cell receptor signaling pathway, and human T-cell leukemia virus 1 (HTLV-1) infection. 

**Figure 5:**
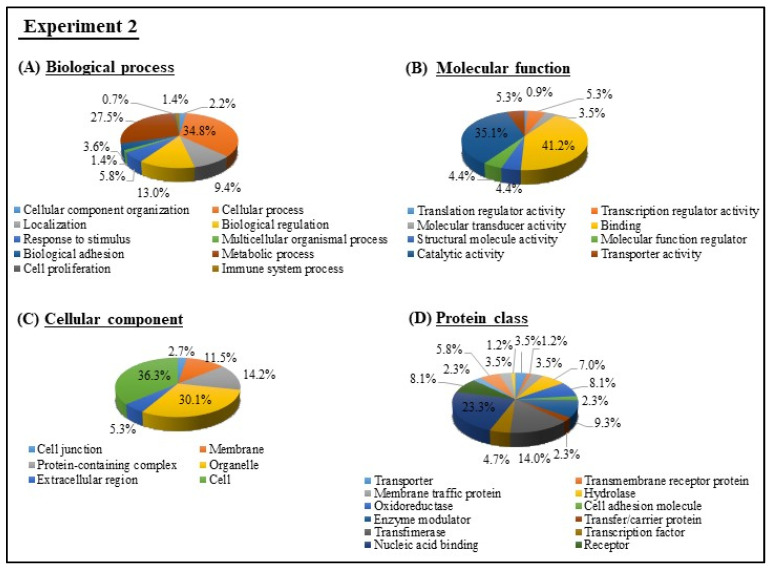
GO of DEGs in milk EVs from BLV-infected cattle with HPL + HLDH in Experiment 2 were analyzed and categorized according to biological processes (**A**), molecular functions (**B**), cellular components (**C**), and protein class (**D**), using PANTHER analysis.

GO analysis was performed for the 176 DEGs in milk EVs from BLV-infected cattle with HPL + HLDH in Experiment 2 (Figure 5). The most abundant biological processes included cellular process (34.8%) and metabolic process (27.5%). The most prevalent molecular functions were binding (41.2%) and catalytic activity (35.1%). Cellular components were mostly engaged in cell (36.3%) and organelle (30.1%). The most enriched protein class included nucleic acid-binding proteins (23.3%), membrane traffic protein (14), and receptors (8.1%).

**Figure 6:**
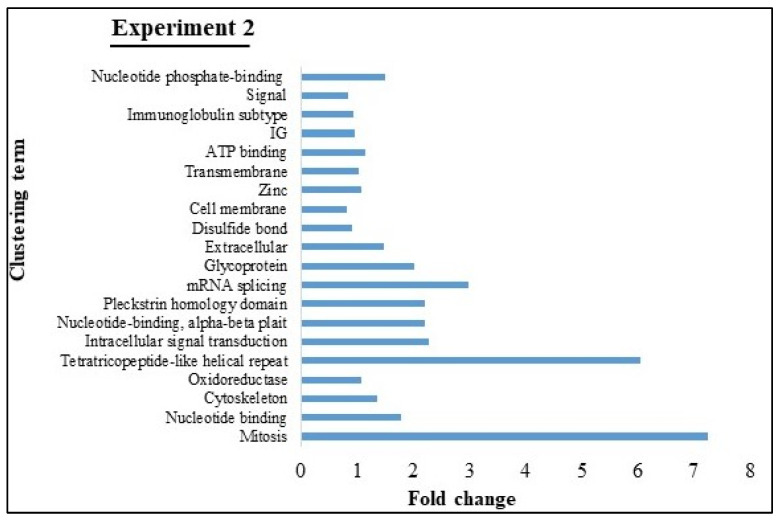
Functional annotation clustering (using DAVID v6.8) of DEGs enriched in milk EVs from BLV-infected cattle with HPL + HLDH in Experiment 2 (top 20 with high fold change). X-axis represented fold change and Y-axis represented clustering term.

Functional annotation clustering identified a total of 20 clusters, including mitosis, tetratricopeptide-like helical repeat, mRNA splicing, glycoprotein, extracellular, nucleotide-binding, and so on (Figure 6). The KEGG pathway identified 197 distinct classes of pathways of DEGs from Experiment 2 (Table 3). Based on these results, the top 10 pathways included metabolic pathways, pathways in cancer, human papillomavirus infection, protein processing in endoplasmic reticulum, influenza A virus infection, chronic myeloid leukemia, HTLV-1 infection, viral carcinogenesis, human immunodeficiency virus 1 (HIV-1) infection, and Rap 1 signaling pathway.

## 4. Discussion

In the current study, we aimed to provide a new insight into the mRNA profile in milk EVs from BLV-infected cattle. Our present study identified 75 and 176 DEGs in Experiments 1 and 2, respectively, without any overlap within these experiments. The host pathophysiological conditions, experimental variation, course of infection, and other unknown factors, would explain the differences in DETs in these two experiments. The current study used two distinct sample sets such as milk EVs from BLV-infected cattle with HPL (Experiment 1) or with HPL + HLDH (Experiment 2), therefore the level of LDH would be the one of the influencing factors. Results indicated that during the disease progression in host, both cellular and genomic information were altered according to the infection advancement. Another explanation could be that host-virus interaction possibly followed a multi-dimensional process; while viruses attempted to take over the cellular functions for their own advantage, gene expression of the host cell got altered. A previous study had reported that, like other retroviruses, HIV-1 infection causes profound effects on EV-mediated cells through multiple mechanisms [26]. Another study had reported that oncogenic viruses, as well as others that are able to establish long-term persistent infections, could alter the contents of EVs for facilitating infection, thereby contributing to persistence and pathogenesis [27]. 

Based on these results, many DEGs have been identified, which play an important role in tumor/cancer progression, cellular invasion, and immunosuppression. Previous reports had demonstrated overexpression of *LAMC2* to be related to multiple types of cancer progression, migration, and invasion in humans [28,29]. *BOLA-DQB* encoded a major histocompatibility complex, whose expression was altered in malignant cancers in humans; *NYNRIN* was shown to be a predisposing gene for Wilms tumor that causes common renal cancer in children [30,31]. Overexpression of *MYOF* has been associated with many cancers in humans, including breast cancer, lung cancer, and pancreatic cancer, and is shown to promote tumorigenesis, tumor cell motility, proliferation, migration, and metastasis [32,33,34]. Furthermore, other studies had reported up-regulation or down-regulation of *TMEM156*, *ELOVL5*, *KIF23*, *AMPD3*, *CCNB1*, *CENPE*, *UBE2C*, *NXPE3*, and *PDE4DIP* to be associated with numerous types of cancers in humans, such as breast cancer, ovarian cancer, non-small cell lung cancer, clear cell renal cell carcinoma, stomach cancer, colorectal cancer, esophageal cancer, pancreatic cancer, hepatocellular cancer, lung cancer, and prostate cancer [35,36,37,38,39,40,41,42,43,44,45]. Our results were consistent with the previous findings that indicated DEGs to possibly have great implication in tumor/cancer progression. 

In this study, the DEGs in milk EVs from BLV-infected cattle were classified based on several bioinformatics analysis tools and provided useful data for better understanding of BLV infection progression. From GO analysis, DEGs in milk EVs from BLV-infected cattle were revealed to be meaningfully involved in major molecular functions, biological processes, and cellular components, and were categorized in distinct protein classes including cellular processes, metabolic processes, response to stimuli, cells, catalytic activity, binding, extracellular regions, and so on. These findings were consistent with those from previous studies, which had revealed EVs from many tumors/cancers to be engaged in various biological functions, such as immune modulatory features, protein binding, signal transduction, and so on [46,47,48]. The previous study reported that during BLV infection, gene involved in diverse biological procedure including cellular proliferation, innate immune response, and metabolic process [49]. Functional annotation clustering indicated that during BLV infection progression, the mitotic activity of cells may be increased, altering the immune activities, mRNA splicing, and involving intracellular signal transduction. In particular, the most significant KEGG pathways were associated with cancer pathways, metabolic pathways, viral infection pathways, Ras and Rap 1 signaling pathways, and so on. Li et al. [36] had reported genes from rhabdomyosarcoma to be enriched in many pathways, such as metabolism, viral infectious diseases, immune system, signal transduction, HTLV-1 infection, herpes simplex virus infection, Epstein-Barr virus infection, and cancer. GO terms in the present study indicated BLV infection to mainly modify host metabolism, cellular components, signal transduction, and cell proliferation, and mRNA profile in milk EVs reflected these modifications. There were few limitations to the present study. First, the experimental data came from a small number of groups of animals. Second, our DEGs found in both Experiments 1 and 2 were not validated by qRT-PCR. Third, due to the sampling and experimental differences, the current study did not find any overlap genes involved in leukemogenesis or lymphoma formation unlike previous studies [50,51]. To overcome these limitations, we need to obtain data from a large number of animal groups and more experiments are required to validate those DEGs in milk EVs from BLV-infected cattle.

In summary, the current study utilized microarray analysis and GO and KEGG analyses to explore the distinct number of DEGs in milk EVs from BLV-infected cattle. The DEGs from both experiments showed profound importance in cancer progression and metabolic processes. In addition, our results also provided a promising resource for the development of biomarkers for BLV progression. Further experiments would be required to validate these DEGs for discovering specific biomarkers of BLV infection and progression. To the best of our knowledge, this is the first report describing the mRNA profile in milk EVs from BLV-infected cattle. 

## Figures and Tables

**Table 1:** Assessment of BLV infection and clinical status of cattle used in this study ^※1^.

	Cattle no.	Age ^※2^ (Month)	ELISA ^※3^ (Antibody)	Nested PCR	Proviral Load ^※4^	WBC ^※5^ (/µL)	Lymphocyte (/µL)	Key of EC ^※6^	LDH ^※7^ Total (IU/l)	LDH Isozyme (%)					
										1	2	3	2 + 3	4	5
**Experiment 1**	**Uninfected cattle**														
	U1	59.0	-	-	NT	NT	NT	NT	NT	NT	NT	NT	NT	NT	NT
	U2	57.7	-	-	NT	NT	NT	NT	NT	NT	NT	NT	NT	NT	NT
	U3	94.8	-	-	NT	NT	NT	NT	NT	NT	NT	NT	NT	NT	NT
	U4	49.8	-	-	NT	NT	NT	NT	NT	NT	NT	NT	NT	NT	NT
	**BLV-infected cattle with HPL** **^※8^**														
	B1	74.0	+	NT	15,405	15,300	12,393	+	NT	NT	NT	NT	NT	NT	NT
	B2	78.5	+	NT	14,571	12,900	8127	+	NT	NT	NT	NT	NT	NT	NT
	B3	107.6	+	NT	13,918	11,200	6720	±	NT	NT	NT	NT	NT	NT	NT
	B4	53.3	+	NT	31,093	16,500	11,880	+	NT	NT	NT	NT	NT	NT	NT
**Experiment 2**	**Uninfected cattle with LLDH** **^※9^**														
	U1	33.6	-	-	NT	7100	2592	-	963	70.6	15.0	9.4	24.4	3.3	1.7
	U2	43.2	-	-	NT	8800	3142	-	729	72.3	14.5	8.0	22.5	3.2	2.0
	U3	37.1	-	-	NT	9700	4113	-	1062	75.6	15.0	6.1	21.1	2.0	1.3
	U4	33.5	-	-	NT	7800	4547	-	821	69.7	12.9	11.0	23.9	4.1	2.3
	**BLV-infected cattle with HPL + HLDH** **^※10^**														
	B1	24.8	+	+	10,264	11,900	6593	-	909	48.1	29.4	15.0	44.4	5.1	2.4
	B2	38.5	+	+	5883	8000	4592	-	901	51.9	25.6	15.4	41.0	5.0	2.1
	B3	23.5	+	+	6550	16,200	8699	-	918	52.4	27.5	14.2	41.7	4.0	1.9
	B4	22.6	+	+	10,781	15,000	8940	-	819	51.4	25.3	15.4	40.7	5.5	2.4

+, positive; -, negative; ±, suspect; NT, not tested; ^※1^ BLV, bovine leukemia virus; ^※2^ Age was at the blood sampling; ^※3^ ELISA, anti-BLV antibody enzyme-linked immunosorbent assay; ^※4^ proviral load was measured by a CycleavePCR Reaction Mix (copies/100 ng of WBC DNA); ^※5^ WBC, white blood cell; ^※6^ Key of EC, leucosis-key of the European Community; ^※7^ LDH, lactate dehydrogenase; ^※8^ HPL, high proviral load; ^※9^ LLDH, low lactate dehydrogenase; ^※10^ HLDH, high lactate dehydrogenase.

**Table 2:** Kyoto Encyclopedia of Genes and Genomes (KEGG) pathways (important 10) of DEGs (up-regulated and down-regulated) in milk EVs from BLV-infected cattle with HPL in Experiment 1.

KEGG Pathway Name	Gene List	Gene No.
Pathways in cancer	AXIN1, LAMC2, PIK3CB, FGF22	4
PI3K-Akt signaling pathway	MYB, LAMC2, PIK3CB, FGF22	4
Metabolic pathways	COX2, PGAM2, PIK3CB, HSD17B6	4
MAPK signaling pathway	CACNA1A, FGF22, PTPN5	3
Cell adhesion molecules	PVRL1, BLA-DQB, IGSF11	3
Ras signaling pathway	PIK3CB, FGF22, RASAL3	3
Phagosome	DYNC1H1, BLA-DQB	2
B cell receptor signaling pathway	CD79A, PIK3CB	2
HTLV-1 infection ※	PIK3CB, BLA-DQB	2

※ Human T-cell leukemia virus-1.

**Table 3:** KEGG pathways (important 10) of DEGs in milk EVs from BLV-infected cattle with HPL + HLDH in Experiment 2.

KEGG Pathway Name	Gene List	Gene No.
Metabolic pathways	NNT, CKMT1B, SCP2, ISYNA1, GNS, NADSYN1, ALDH8A1, SLC33A1, AMPD3, ENTPD4, ELOVL6, DHCR24, MGAT1, ALAS1, ELOVL5	15
Pathways in cancer	BAX, LPAR1, CTBP2, GNAS, TGFBR1, JUP, BAK1, DDB2	8
Human papillomavirus infection	BAX, GNAS, PPP2R5B, BAK1, TBPL1	5
Protein processing in endoplasmic reticulum	BAX, HSPA1A, PRKCSH, EDEM2; ER, BAK1	5
Influenza A	BAX, TLR4, TRIM25, TMPRSS2, BAK1	5
Chronic myeloid leukemia	BAX, CTBP2, TGFBR1, BAK1, DDB2	5
HTLV-1 infection	BAX, ITGB2, TGFBR1, CDC20, TBPL1;	5
Viral carcinogenesis	BAX, BAK1, CDC20, TBPL1	4
HIV-1 infection ※	BAX, TLR4, CCNB1, BAK1	4
Rap1 signaling pathway	LPAR1, GNAS, ITGB2	3

※ Human immunodeficiency virus-1.

## Supplementary Materials

The following are available online at https://www.mdpi.com/1999-4915/12/6/669/s1, Table S1: DEGs (up-regulated and down-regulated) in milk EVs from BLV-infected cattle with HPL in Experiment 1; Table S2: DEGs (up-regulated and down-regulated) in milk EVs from BLV-infected cattle with HPL + HLDH in Experiment 2; and Figure S1: Representatives of morphological features of milk EVs from BLV-infected and uninfected cattle by transmission electron microscopy analysis.
