# Supplementary material for: mRNA Profile in Milk Extracellular Vesicles from Bovine Leukemia Virus-Infected Cattle
Source: Viruses. 2020 Jun 20;12(6):669. doi: 10.3390/v12060669 (PMC7354454; doi:10.3390/v12060669)
Supplement: Supplementary file 1 [file viruses-12-00669-s001.zip › Table S1.docx]

Table S1. Significantly up-regulated and down-regulated of DEGs in milk EVs from BLV-infected cattle with HPL in Experiment 1

| Probe name | Gene symbol | Gene ID | Fold change value | Regulation |
| --- | --- | --- | --- | --- |
| A_73_P119171 | LAMC2 | 511043 | 4.59 | up |
| A_73_P136781 | SLC35E4 | 523783 | 3.65 | up |
| A_73_P266451 | SLC22A17 | 539848 | 3.49 | up |
| A_73_P203702 | STBD1 | 513376 | 3.26 | up |
| A_73_P038156 | TMEM255A | 533569 | 3.05 | up |
| A_73_P084911 | LOC113896297 | 113896297 | 2.96 | up |
| A_73_P084026 | CACNA1A | 282648 | 2.95 | up |
| A_73_P079046 | NYNRIN | 516993 | 2.82 | up |
| A_73_P362616 | BLA-DQB | 539241 | 2.79 | up |
| A_73_103455 | LOC107133160 | 107133160 | 2.66 | up |
| A_73_P493828 | AXIN1 | 504357 | 2.65 | up |
| A_73_P106821 | CALCR | 613317 | 2.65 | up |
| A_73_P054426 | C6H4orf22 | 615662 | 2.64 | up |
| A_73_P395271 | PLEK | 518658 | 2.63 | up |
| A_73_P088996 | SLC35B4 | 613784 | 2.54 | up |
| A_73_P090841 | OR6C1 | 788009 | 2.51 | up |
| A_73_115779 | PTPN5 | 615850 | 2.46 | up |
| A_73_P194127 | LOC109565844 | 109565844 | 2.44 | up |
| A_73_109084 | LOC113891213 | [113891213](https://www.ncbi.nlm.nih.gov/gene/113891213) | 2.37 | up |
| A_73_105610 | GFRA1 | 534801 | 2.37 | up |
| A_73_P347561 | MED12 | 520974 | 2.31 | up |
| A_73_P115471 | SEMA3D | 536417 | 2.26 | up |
| A_73_P076131 | ANO3 | 100139986 | 2.20 | up |
| A_73_P156097 | C8H9orf131 | 100137826 | 2.18 | up |
| A_73_P109866 | CD79A | 281674 | 2.17 | up |
| A_73_105689 | ISLR2 | 617436 | 2.10 | up |
| A_73_P085056 | FGF22 | 519657 | 2.05 | up |
| A_73_104933 | PDILT | 518716 | 1.98 | up |
| A_73_107583 | IGSF11 | 540003 | 1.94 | up |
| A_73_100922 | TRIM71 | 531886 | 1.92 | up |
| A_73_P179622 | LANCL1 | 540559 | 1.92 | up |
| A_73_P036391 | CYLC2 | 281738 | 1.89 | up |
| A_73_104162 | DNHD1 | 617229 | 1.87 | up |
| A_73_104207 | AUNIP | 539251 | 1.84 | up |
| A_73_P126416 | GPR101 | 538781 | 1.79 | up |
| A_73_102941 | COX2 | 3283880 | 1.77 | up |
| A_73_P063896 | GATA2 | 506809 | 1.77 | up |
| A_73_P108216 | MYB | 317776 | 1.77 | up |

Table S1. Continue

| Probe name | Gene symbol | Gene ID | Fold change value | Regulation |
| --- | --- | --- | --- | --- |
| A_73_P048311 | LOC511494 | 511494 | 1.76 | up |
| A_73_P037926 | OMD | 280885 | 1.76 | up |
| A_73_P369066 | GAS7 | 614517 | 1.73 | up |
| A_73_116379 | PNMA2 | 540301 | 1.72 | up |
| A_73_P251446 | HSD17B6 | 533086 | 1.69 | up |
| A_73_P407241 | TMEM94 | 512110 | 1.67 | up |
| A_73_P074111 | CHRNA4 | 537251 | 1.66 | up |
| A_73_111741 | IKZF1 | 541154 | 1.64 | up |
| A_73_100113 | RASAL3 | 540027 | 1.61 | up |
| A_73_101290 | PGAM2 | 515067 | 1.61 | up |
| A_73_P095036 | CATIP | 514380 | 1.60 | up |
| A_73_P075191 | PVRL1 | 533492 | 1.59 | up |
| A_73_118500 | LOC113889514 | [113889514](https://www.ncbi.nlm.nih.gov/gene/113889514) | 1.59 | up |
| A_73_106629 | TNP2 | 281538 | 1.55 | up |
| A_73_P071601 | KCNT2 | 781505 | 1.55 | up |
| A_73_P271851 | DYNC1H1 | 537748 | 1.54 | up |
| A_73_P126251 | GRHL2 | [519918](https://www.ncbi.nlm.nih.gov/gene/519918) | 1.53 | up |
| A_73_P051176 | ADGRL1 | 788252 | 1.49 | up |
| A_73_P133721 | IQCE | 618833 | 1.47 | up |
| A_73_P128421 | TRERF1 | 504797 | 1.47 | up |
| A_73_P111906 | NSG1 | 523110 | 1.46 | up |
| A_73_P387426 | MFSD14B | 510635 | 1.44 | up |
| A_73_P354216 | JADE2 | [113895358](https://www.ncbi.nlm.nih.gov/gene/113895358) | 1.41 | up |
| A_73_P134451 | GXYLT1 | [113893009](https://www.ncbi.nlm.nih.gov/gene/113893009) | 1.35 | up |
| A_73_P086606 | ENTPD4 | [113897304](https://www.ncbi.nlm.nih.gov/gene/113897304) | 1.33 | up |
| A_73_P063121 | CTDSPL | 538425 | 1.31 | up |
| A_73_P034181 | TFF2 | 616105 | 10.22 | down |
| A_73_P035256 | DKK2 | 541161 | 8.07 | down |
| A_73_P032531 | SERPINB9 | 524513 | 2.25 | down |
| A_73_P363601 | OLFML2B | 513053 | 1.98 | down |
| A_73_P072011 | NOL9 | 523474 | 1.86 | down |
| A_73_P049341 | FAM151B | 535260 | 1.51 | down |
| A_73_P321181 | NKRF | 100124519 | 1.50 | down |
| A_73_P387316 | PIK3CB | 517948 | 1.46 | down |
| A_73_118712 | CYHR1 | 282612 | 1.43 | down |
| A_73_104051 | WDR12 | 528209 | 1.33 | down |
| A_73_P375401 | FBXW12 | 528919 | 1.30 | down |
