# Supplementary material for: mRNA Profile in Milk Extracellular Vesicles from Bovine Leukemia Virus-Infected Cattle
Source: Viruses. 2020 Jun 20;12(6):669. doi: 10.3390/v12060669 (PMC7354454; doi:10.3390/v12060669)
Supplement: Supplementary file 1 [file viruses-12-00669-s001.zip › Table S2.docx]

Table S2. Significantly up-regulated and down-regulated of DEGs in milk EVs from BLV-infected cattle with HPL+HLDH in Experiment 2

| Probe name | Gene symbol | Gene ID | Fold change value | Regulation |
| --- | --- | --- | --- | --- |
| A_73_P415681 | MYOF | 513597 | 12.6696 | up |
| A_73_P031496 | ELOVL5 | 617293 | 12.5594 | up |
| A_73_112371 | TMEM156 | 533681 | 10.7884 | up |
| A_73_P074346 | KIF23 | 515748 | 7.28765 | up |
| A_73_P033076 | AMPD3 | 519803 | 6.53952 | up |
| A_73_103826 | CCNB1 | 327679 | 6.39658 | up |
| A_73_P050701 | CENPE | 281681 | 5.74506 | up |
| A_73_100039 | UBE2C | 506962 | 5.61198 | up |
| A_73_P068891 | NXPE3 | 532838 | 5.3169 | up |
| A_73_121055 | PDE4DIP | 508547 | 4.70572 | up |
| A_73_P033971 | TSPAN2 | 539001 | 4.19931 | up |
| A_73_P285906 | ANLN | 518274 | 3.8064 | up |
| A_73_P321781 | ELOVL6 | 533333 | 3.77583 | up |
| A_73_100019 | CHIC1 | 615065 | 3.73917 | up |
| A_73_P159712 | CDC20 | 515376 | 3.6939 | up |
| A_73_P044806 | MAP9 | 613821 | 3.50708 | up |
| A_73_P044856 | ABR | 515556 | 3.2325 | up |
| A_73_106147 | PDK3 | 510841 | 3.16915 | up |
| A_73_P045331 | SPC24 | 509117 | 3.13963 | up |
| A_73_P044236 | CALU | 539218 | 3.03805 | up |
| A_73_117737 | GDPD1 | 615890 | 2.87672 | up |
| A_73_119135 | POSTN | 281960 | 2.86479 | up |
| A_73_P120086 | ARHGEF2 | 505940 | 2.73601 | up |
| A_73_P052701 | GPR161 | 768006 | 2.7022 | up |
| A_73_116565 | MIS18A | 510809 | 2.53376 | up |
| A_73_P036741 | MPDZ | 536863 | 2.47664 | up |
| A_73_P442746 | ZDHHC2 | 536310 | 2.4347 | up |
| A_73_102903 | PTPN20 | 517883 | 2.40094 | up |
| A_73_P508098 | SLC43A2 | 511955 | 2.40022 | up |
| A_73_P239305 | TMEM59 | 509775 | 2.38133 | up |
| A_73_P327926 | TTPAL | 526582 | 2.34314 | up |
| A_73_115262 | EDEM2 | 513253 | 2.27697 | up |
| A_73_117605 | ARMCX6 | 768308 | 2.10962 | up |
| A_73_P347011 | ABHD16A | 513252 | 2.08633 | up |
| A_73_P386991 | LPAR1 | 281136 | 1.98695 | up |
| A_73_P349171 | GNS | 512444 | 1.93156 | up |
| A_73_P438021 | ITGB2 | 281877 | 1.91975 | up |

Table S2. Continue

| Probe name | Gene symbol | Gene ID | Fold change value | Regulation |
| --- | --- | --- | --- | --- |
| A_73_P033466 | LMAN2L | 539289 | 1.91914 | up |
| A_73_113076 | TPX2 | 507226 | 1.84229 | up |
| A_73_116813 | LOC109576896 | 1.1E+08 | 1.84104 | up |
| A_73_112711 | BSG | 508716 | 1.79624 | up |
| A_73_P066656 | TMEM19 | 538974 | 1.76531 | up |
| A_73_106851 | STX2 | 519148 | 1.76437 | up |
| A_73_P090416 | NNT | 280878 | 1.76419 | up |
| A_73_P329836 | CKMT1B | 281692 | 1.75426 | up |
| A_73_106315 | SEC22C | 614905 | 1.7473 | up |
| A_73_P227212 | LOC531038 | 531038 | 1.72483 | up |
| A_73_102373 | TSPAN15 | 522371 | 1.69668 | up |
| A_73_P045921 | PDZD8 | 519573 | 1.6908 | up |
| A_73_105849 | IFT140 | 1E+08 | 1.68285 | up |
| A_73_P256331 | AURKA | 504437 | 1.68069 | up |
| A_73_P465973 | TMPRSS2 | 511037 | 1.67728 | up |
| A_73_P048721 | ENTPD4 | 531411 | 1.67298 | up |
| A_73_110296 | ATRN | 281017 | 1.66479 | up |
| A_73_116206 | HAPLN3 | 515224 | 1.63466 | up |
| A_73_P439506 | SLC33A1 | 517797 | 1.62556 | up |
| A_73_119085 | KCNK1 | 505563 | 1.62259 | up |
| A_73_117528 | TMEM127 | 513620 | 1.61768 | up |
| A_73_P451916 | CACNA1A | 282648 | 1.59737 | up |
| A_73_102196 | ZNF318 | 786496 | 1.58215 | up |
| A_73_P260451 | RFLNB | 1.1E+08 | 1.57711 | up |
| A_73_P284236 | COMTD1 | 514949 | 1.5643 | up |
| A_73_101073 | DDHD1 | 338047 | 1.53247 | up |
| A_73_P088621 | GNAS | 281793 | 1.50426 | up |
| A_73_P092281 | CCDC159 | 785202 | 1.50354 | up |
| A_73_P180212 | MTX3 | 616872 | 1.48524 | up |
| A_73_P289031 | GNPTAB | 509610 | 1.48517 | up |
| A_73_109550 | LOC404061 | 404061 | 1.47252 | up |
| A_73_P102236 | TLR4 | 281536 | 1.45849 | up |
| A_73_P153036 | PCSK7 | 515398 | 1.43578 | up |
| A_73_115691 | BAX | 280730 | 1.43174 | up |
| A_73_108146 | BAK1 | 514090 | 1.42228 | up |
| A_73_114599 | TGFBR1 | 282382 | 1.42198 | up |
| A_73_117489 | DHCR24 | 533726 | 1.40675 | up |
| A_73_P087236 | LOC113881225 | 1.1E+08 | 1.38592 | up |

Table S2. Continue

| Probe name | Gene symbol | Gene ID | Fold change value | Regulation |
| --- | --- | --- | --- | --- |
| A_73_P288606 | LYRM7 | 618451 | 1.37096 | up |
| A_73_107676 | TAPT1 | 615598 | 1.36985 | up |
| A_73_P301611 | AIFM1 | 535714 | 1.356 | up |
| A_73_P445746 | SLC11A2 | 521189 | 1.3537 | up |
| A_73_118052 | KDM1B | 513667 | 1.34391 | up |
| A_73_P258096 | MGAT1 | 534248 | 1.3412 | up |
| A_73_114582 | THNSL1 | 788561 | 1.33108 | up |
| A_73_105768 | SURF4 | 526045 | 1.32218 | up |
| A_73_110544 | ALAS1 | 534286 | 1.32163 | up |
| A_73_P033231 | TMEM41A | 505368 | 1.3188 | up |
| A_73_110467 | TBPL1 | 613818 | 1.31539 | up |
| A_73_P040376 | KIDINS220 | 536267 | 1.31233 | up |
| A_73_118890 | RCN2 | 512717 | 1.29503 | up |
| A_73_119160 | LSM12 | 538005 | 1.29455 | up |
| A_73_P178572 | RNF126 | 507447 | 1.28267 | up |
| A_73_120320 | RC3H2 | 783330 | 1.28021 | up |
| A_73_P033411 | SLC15A4 | 510499 | 1.26462 | up |
| A_73_P309455 | OTULIN | 508963 | 1.26389 | up |
| A_73_105471 | DCAF16 | 777600 | 1.26123 | up |
| A_73_106579 | PLEKHM2 | 515552 | 1.25984 | up |
| A_73_P311171 | KIAA2013 | 506454 | 1.24744 | up |
| A_73_P405761 | CD36 | 281052 | 1.24442 | up |
| A_73_P478133 | ERLIN1 | 617074 | 1.24381 | up |
| A_73_117673 | PPP2R5B | 505915 | 1.22395 | up |
| A_73_116757 | MERTK | 504429 | 3.24929 | down |
| A_73_105174 | FETUB | 504615 | 2.57967 | down |
| A_73_P034011 | HSPA1B | 282254 | 2.36487 | down |
| A_73_P143106 | NEUROD4 | 519656 | 2.36115 | down |
| A_73_117309 | LOC616782 | 616782 | 2.23945 | down |
| A_73_P292536 | GPRC5A | 516026 | 2.21788 | down |
| A_73_111258 | MED24 | 504613 | 1.99683 | down |
| A_73_103155 | DHX58 | 508378 | 1.9625 | down |
| A_73_P048176 | ZMIZ1 | 616740 | 1.9625 | down |
| A_73_P251081 | ISYNA1 | 509394 | 1.93104 | down |
| A_73_P149571 | CCDC82 | 511627 | 1.8709 | down |
| A_73_112433 | RGS9 | 281453 | 1.70328 | down |
| A_73_P058926 | PRKCSH | 338067 | 1.63268 | down |
| A_73_105811 | CHD2 | 535026 | 1.58808 | down |

Table S2. Continue

| Probe name | Gene symbol | Gene ID | Fold change value | Regulation |
| --- | --- | --- | --- | --- |
| A_73_P150781 | EEF1D | 516473 | 1.58706 | down |
| A_73_P278191 | TERF1 | 519474 | 1.58178 | down |
| A_73_P411556 | RGS7 | 281452 | 1.55927 | down |
| A_73_P345936 | RPL4 | 510547 | 1.54251 | down |
| A_73_P446126 | NADSYN1 | 513400 | 1.53637 | down |
| A_73_P337541 | GRAMD1C | 505581 | 1.48965 | down |
| A_73_108349 | ALDH8A1 | 513537 | 1.47596 | down |
| A_73_P036793 | NOP53 | 506401 | 1.47368 | down |
| A_73_P050626 | POLE2 | 518653 | 1.45568 | down |
| A_73_P284786 | BPIFB3 | 522020 | 1.45515 | down |
| A_73_118250 | BRD3 | 525051 | 1.42003 | down |
| A_73_P078341 | RFC4 | 504637 | 1.41886 | down |
| A_73_108361 | SOD1 | 281495 | 1.41386 | down |
| A_73_P040721 | C11H2orf68 | 616899 | 1.40217 | down |
| A_73_112870 | TNRC18 | 526616 | 1.4012 | down |
| A_73_P266171 | TBC1D14 | 618286 | 1.39843 | down |
| A_73_P042691 | ATP5S | 493709 | 1.39355 | down |
| A_73_P044261 | TMPO | 510267 | 1.38781 | down |
| A_73_114828 | VEZT | 535351 | 1.3772 | down |
| A_73_113379 | STX19 | 514487 | 1.37439 | down |
| A_73_P091006 | PABPC1 | 282296 | 1.36609 | down |
| A_73_P259251 | CSRP1 | 615329 | 1.36451 | down |
| A_73_P038911 | NBN | 522943 | 1.36397 | down |
| A_73_P034666 | PEX14 | 507393 | 1.36385 | down |
| A_73_P450591 | STAG1 | 539234 | 1.3616 | down |
| A_73_P253201 | GPANK1 | 617553 | 1.35283 | down |
| A_73_104739 | POLA2 | 514793 | 1.35094 | down |
| A_73_P395436 | ZSWIM7 | 514094 | 1.34131 | down |
| A_73_103900 | IL17RC | 504855 | 1.33088 | down |
| A_73_P074658 | TCF25 | 1E+08 | 1.33036 | down |
| A_73_P092606 | JUP | 445543 | 1.32323 | down |
| A_73_121460 | TRIM25 | 510923 | 1.3206 | down |
| A_73_100980 | NASP | 512427 | 1.31738 | down |
| A_73_P038246 | OTUD1 | 1E+08 | 1.31625 | down |
| A_73_P422051 | FRMD6 | 530910 | 1.31283 | down |
| A_73_104115 | GLE1 | 540982 | 1.31283 | down |
| A_73_P441976 | PSMB1 | 514237 | 1.31171 | down |
| A_73_P043151 | HNRNPH3 | 539850 | 1.30784 | down |
| A_73_P470368 | PUM1 | 535450 | 1.30573 | down |

Table S2. Continue

| Probe name | Gene symbol | Gene ID | Fold change value | Regulation |
| --- | --- | --- | --- | --- |
| A_73_P293046 | GSE1 | 538506 | 1.30541 | down |
| A_73_P060436 | XRCC1 | 616905 | 1.30421 | down |
| A_73_P117866 | JADE3 | 506664 | 1.29911 | down |
| A_73_114694 | C20H5orf34 | 514072 | 1.29188 | down |
| A_73_P318791 | SWAP70 | 533720 | 1.29178 | down |
| A_73_P256711 | CTBP2 | 281730 | 1.289 | down |
| A_73_P064966 | CCHCR1 | 514215 | 1.28743 | down |
| A_73_P046281 | RPAP3 | 510621 | 1.28373 | down |
| A_73_P031696 | SNRPA1 | 512584 | 1.27956 | down |
| A_73_114994 | NEDD1 | 519463 | 1.27458 | down |
| A_73_102653 | ZCCHC6 | 513209 | 1.27407 | down |
| A_73_P113611 | EMC2 | 616813 | 1.26912 | down |
| A_73_P116316 | DDB2 | 519357 | 1.25989 | down |
| A_73_P073711 | SCP2 | 508918 | 1.25868 | down |
| A_73_105309 | TFIP11 | 509349 | 1.25717 | down |
| A_73_P469998 | CTDSP2 | 506115 | 1.25642 | down |
| A_73_107683 | RBM5 | 534216 | 1.2558 | down |
| A_73_P368221 | PRPF39 | 505547 | 1.2552 | down |
| A_73_P269446 | HMGN5 | 767875 | 1.25445 | down |
| A_73_109458 | PKP3 | 516247 | 1.25388 | down |
| A_73_P033601 | U2SURP | 505019 | 1.25048 | down |
| A_73_P074571 | ARMC8 | 539591 | 1.24208 | down |
| A_73_108505 | FAM45A | 534370 | 1.22892 | down |
| A_73_P375486 | ZNF638 | 517669 | 1.22387 | down |
