# Supplementary figures and images for: mRNA Profile in Milk Extracellular Vesicles from Bovine Leukemia Virus-Infected Cattle
Source: Viruses. 2020 Jun 20;12(6):669. doi: 10.3390/v12060669 (PMC7354454; doi:10.3390/v12060669)

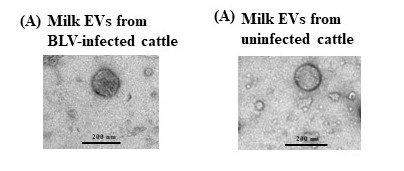

Supplement: Supplementary file 1 [file viruses-12-00669-s001.zip › Figure S1.jpg]
